# The complete chloroplast genome of *Prunus verecunda*, and phylogenetic analysis with amygdaleae species

**DOI:** 10.1080/23802359.2019.1673228

**Published:** 2019-10-04

**Authors:** Dongyue Jiang, Xinhong Liu, Xin Shen

**Affiliations:** Zhejiang Academy of Forestry, Hangzhou, PR China

**Keywords:** Prunus verecunda, chloroplast genome, pair-end sequencing, phylogenetic analysis

## Abstract

We assembled complete chloroplast (cp) genome of *Prunus verecunda* based on Illumina sequencing. The cp genome of *P. verecunda* is 157,917 bp in length with 129 genes comprising 84 protein-coding genes, 37 tRNA genes and 8 rRNA ribosomal genes. The overall GC content of cp genome is 36.7%. A maximum likelihood phylogenetic analysis revealed that *P. verecunda* is sister to *P. serrulata* var. *spontanea* and *P. maximowiczii*.

*Prunus verecunda* Koehne (Rosaceae), also named *P. leveilleana*, is a deciduous tree native to Korea and Japan. This species is considered to be part of *P. serrulata* complex with *P. jamasakura*, which is surrounded taxonomic confusion because of the high degree of morphological intergradation (Chang et al. 2007). It generally has autumnal leaves of reddish-brown or crimson red color and flowers of pale pink to white colors (Iwatsuki et al. 2001). Here, we assembled the complete chloroplast (cp) genome of *P. verecunda* (MN242946) and analyzed its phylogenetic status in Amygdaleae which will be beneficial to future studies on the conservation, phylogeny and breeding of *Cerasus*.

We collected fresh leaves of *P. verecunda* from Wuhan Faya Garden Group Co., Ltd., Hubei province, China (30°29′14.8″N 114°33′1.7″E). A voucher specimen (JDYY061) was deposited in the Herbarium of Zhejiang Academy of Forestry, Hangzhou, China. Total DNA was isolated using Hi-DNAsecure Plant Kit (Tiangen, Beijing, CN). The sequencing DNA from short-insert (<800 bp) paired-end library was performed on a HiSeq XTM Ten analyzer (Illumina, San Diego, California, USA) at Beijing Genomics Institute (BGI, Shenzhen, China). The chloroplast DNA sequences were manually mapped to the reference *P. maximowiczii* (KP760071) and assembled using Geneious (R10.2.3) and SPAdes software (Bankevich et al. 2012; Kearse et al. 2012). The cp genome annotation and correction were implemented using Plann and Sequin (Huang and Cronk 2015).

The *P. verecunda* chloroplast genome is 157,917 bp in length and contains a pair of inverted repeat (IR, 26,437 bp) regions, separating a small single copy (SSC, 19,121 bp) region and a large single copy (LSC, 85,922 bp) region. The whole cp genome encodes 112 unique genes, of which 18 are duplicated in the IRs, giving a total of 129 genes, including 84 protein-coding genes (PCG), 37 tRNA genes, 8 rRNA ribosomal genes. Among these genes, most of genes occur in a single copy, while 6 PCGs, 7 tRNA genes, and 4 rRNA genes in IR regions are duplicated. The overall GC content of *P. verecunda* cp genome is 36.7% with 34.6, 30.2 and 42.5% GC in the LSC, SSC and IR regions, respectively.

To clarify the phylogenetic position of *P. verecunda*, the complete cp genome sequence of *P. verecunda* was used to construct the phylogenetic tree with 18 Amygdaleae species of which cp genome sequences were downloaded from GenBank database. The maximum likelihood (ML) was performed using PhyloSuite (Zhang et al. 2018) with 1000 bootstrap replicates. The phylogenetic tree is comprised of three clades, subg. *Prunus*, subg. *Padus* and subg. *Cerasus*, and *P. verecunda* is sister to *P. serrulata* var. *spontanea* and *P. maximowiczii* (Figure 1).

**Figure 1.**
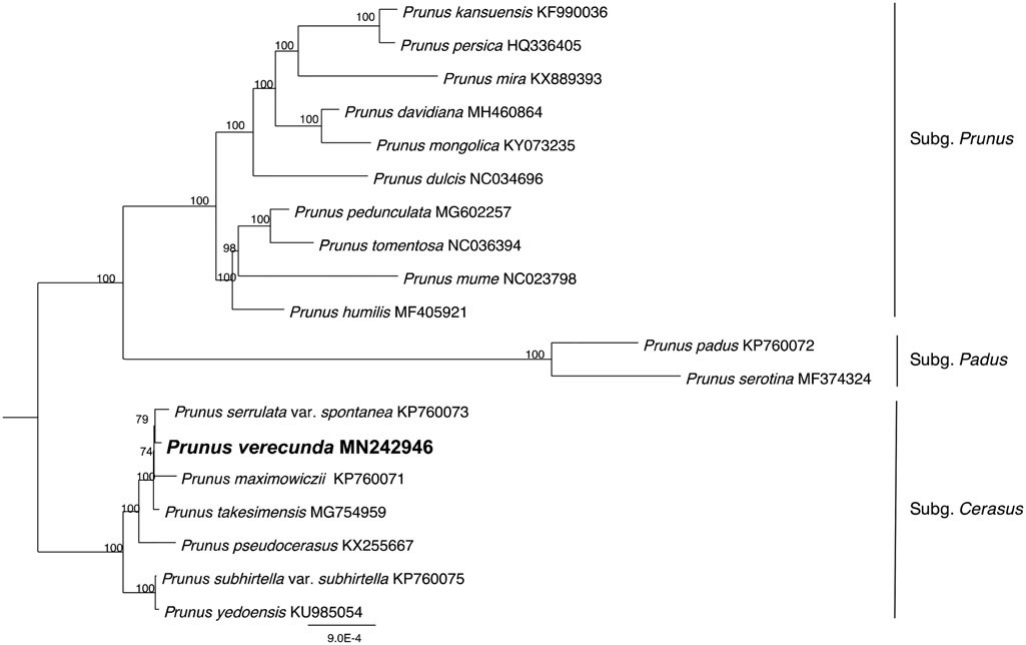
Maximum likelihood phylogenetic tree for *Prunus verecunda* based on 19 complete cp genomes with 1000 bootstrap replicates.
